# Right atrial mass in a patient with COVID‐19 pneumonia: A case report

**DOI:** 10.1002/ccr3.4220

**Published:** 2021-05-15

**Authors:** Fahmi Othman, Abdul Rehman Abid, Sabir Abdulkarim, Mohamad Y. Khatib, Abdulqadir J. Nashwan, Maryam Alkuwari, Abdulwahid AlMulla, Awad Alqahtani

**Affiliations:** ^1^ Department of Cardiology and Cardiovascular Surgery Heart Hospital Hamad Medical Corporation Doha Qatar; ^2^ Critical Care and Pulmonary Medicine Hamad Medical Corporation Doha Qatar; ^3^ Department of Nursing Education and Research Hamad Medical Corporation Doha Qatar; ^4^ Department of Radiology Hamad Medical Corporation Doha Qatar

**Keywords:** atrial mass, benign cardiac tumor, COVID‐19, interatrial septal hypertrophy, lipomatous hypertrophy

## Abstract

Lipomatous hypertrophy of the interatrial septum can have an atypical appearance by transthoracic echocardiography. The authors emphasize on the importance of the multimodality imaging approach to reach the appropriate diagnosis in such cases.

## Graphical abstract

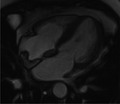

## 1 BACKGROUND

Lipomatous hypertrophy of the interatrial septum (LHIAS) is a benign cardiac tumor. Differential diagnosis of LHIAS consists of atrial masses such as myxomas or lipomas. Herein, we report a 66‐year‐old man, admitted with a case of severe COVID‐19, and were found to have a LHIAS extending to the crista terminalis.

Lipomatous hypertrophy of the interatrial septum (LHIAS) is a well‐described benign cardiac tumor. LHIAS is a quite frequent finding seen by echocardiography. It is estimated to be presented in up to 8% of the general population.^1^

Lipomatous hypertrophy of the interatrial septum is typically diagnosed by transthoracic echocardiography (TTE). However, in some cases, it may have an atypical appearance in TTE images, especially when the echo images are suboptimal.

Differential diagnosis consists of atrial masses such as myxomas or lipomas.^2^

Interatrial septum thickness of 6 mm in the general population and 7mm in the elderly is considered normal.^3^ Lipomatosis has been defined as when the interatrial septum thickness is greater than 15 mm.^4^ Adipose tissue can also accumulate in the subepicardium, crista terminalis, endocardium, and mediastinum.

## 2 CASE PRESENTATION

This is a case of a 66‐year‐old man with a previous history of hypertension, diabetes, chronic kidney disease, and psoriasis. He presented to the communicable disease center (CDC) with a three‐day history of fever, cough, and shortness of breath. On admission, his vital signs were as follows: blood pressure: 115/52 mm Hg, heart rate: 75 b/min, respiratory rate: 20 b/min, and oxygen saturation (O_2_sat): 93% on room air. His body mass index (BMI) was 29 kg/m^2^. Physical examination was remarkable for bilateral chest crackles with normal cardiovascular examination.

His electrocardiogram revealed a sinus rhythm with right bundle branch block and left axis deviation. Chest X‐ray scan showed patchy peripheral ground‐glass opacities in both lungs and soft consolidations in the right middle lobe. Polymerase chain reaction was performed on a nasopharyngeal swab and returned positive for SARS‐CoV‐2. In addition, blood tests showed a WBC count of 5.9 × 10^3^/μL, hemoglobin of 10 gm/dL (N [13‐17 gm/dL]), C‐reactive protein of 146 mg/L [N < 6 mg/L]), ferritin of 2619 ug/L [N < 553 ug/L]), creatinine of 190 μmol/L (N [62‐106 μmol/L]), and D‐dimer of 2.3 mg/L FEU [N < 0.5 mg/L FEU]).

Initially, he was given oxygen therapy at 2 liters per minute via a nasal cannula, but shortly after admission, he became severely hypoxic, and then, he was transferred to the medical intensive care unit and kept on continuous positive airway pressure to maintain oxygen saturation of 95%.

**FIGURE 1:**
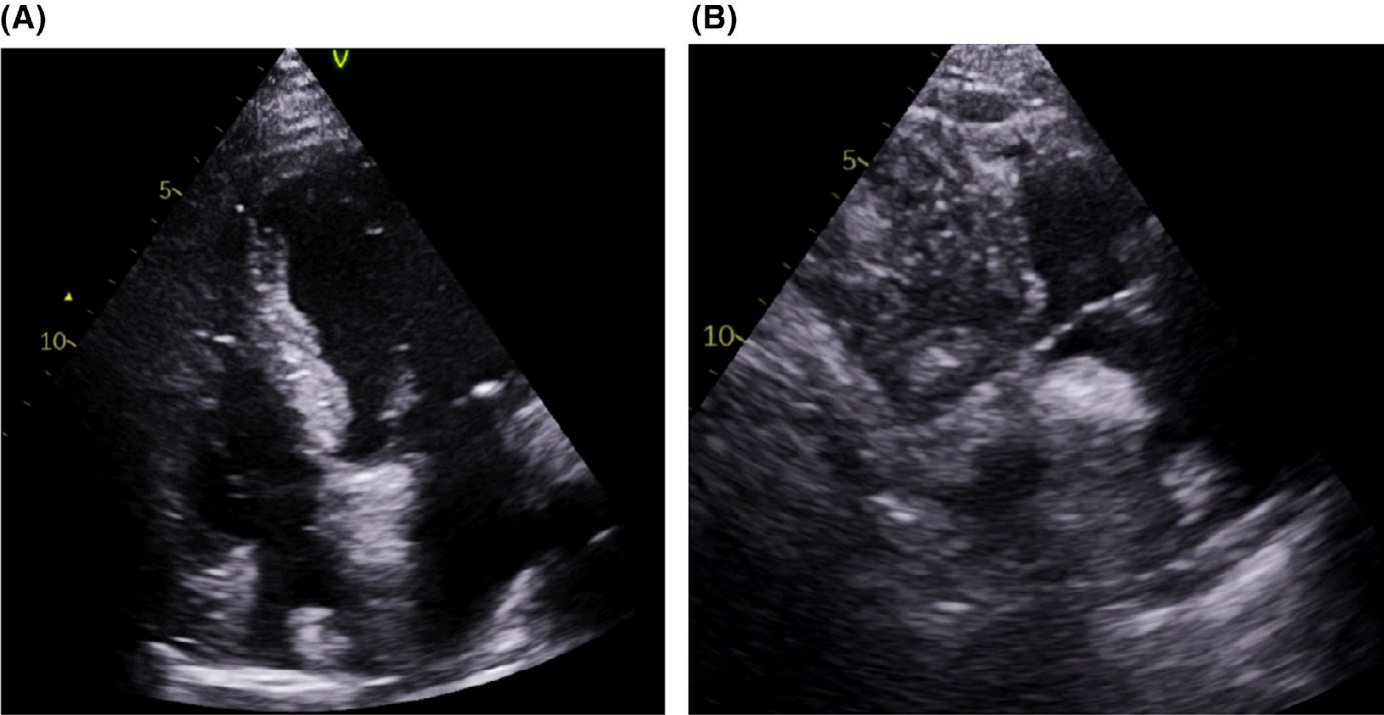
Transthoracic echocardiography (TTE). A, Modified apical four‐chamber view showing a right atrial mass (arrow). B, Right ventricle inflow view showing a large mass protruding into the tricuspid valve annulus (arrowhead)

Transthoracic echocardiography (TTE) was performed, which revealed normal biventricular size and function with no significant valvular disease. Mild pulmonary hypertension with an estimated systolic pulmonary artery pressure of 48 mm Hg was noted. The study showed echo dense mass located on the right atrial (RA) roof, best seen in the four‐chamber view. There was another echo‐lucent mass in the RA protruding into the tricuspid valve annulus, best seen in the RV inflow view, as shown in Figure 1. A prior TTE report that had been performed in another center one month ago revealed no interatrial mass.

Because of acute COVID‐19 pneumonia and elevated D‐dimer, the RA mass was considered and treated as a thrombus. Initially, he was given a therapeutic dose of intravenous heparin and warfarin; thus, the INR was maintained in the therapeutic range between 2 and 3. Repeated echo did not show any significant differences. For a better assessment of the RA mass, transesophageal echocardiography (TEE) was planned, but was not performed due to acute respiratory distress with low oxygen saturation. Instead, 12 days later, upon clinical recovery a cardiac magnetic resonance imaging (CMR) was performed.

**FIGURE 2:**
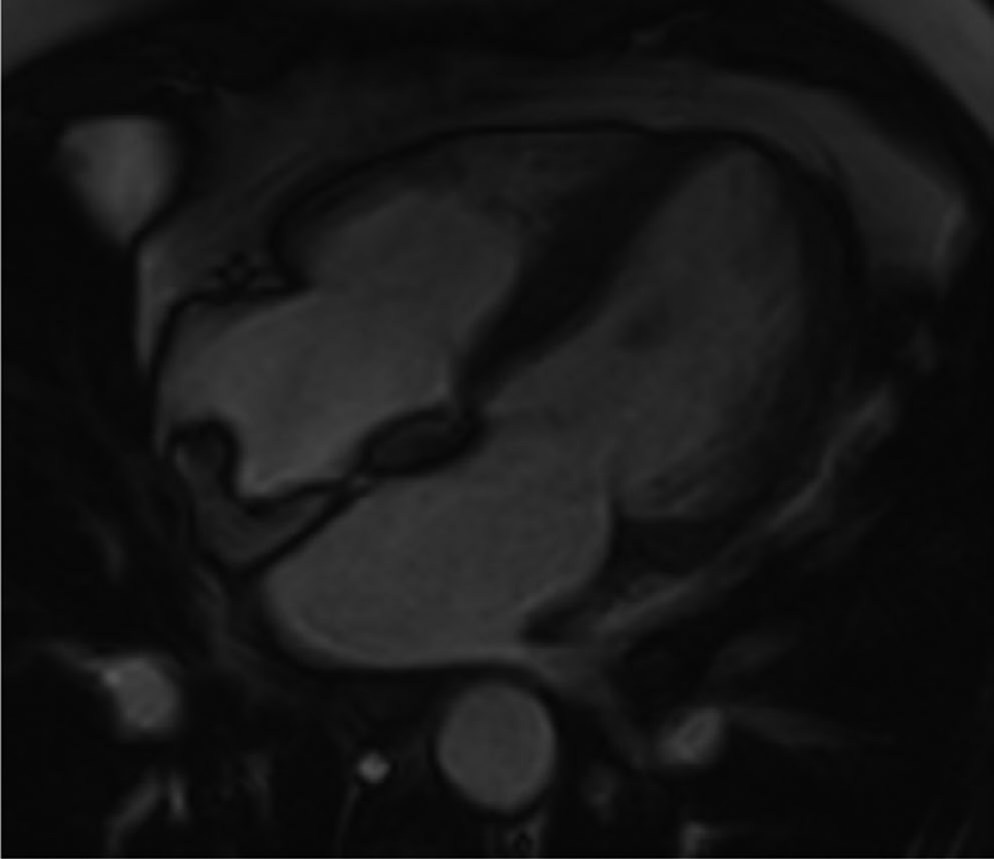
Cardiac magnetic resonance imaging, four‐chamber cine view confirmed the structure attached to the right atrial posterior wall and extends to the interatrial septum

**FIGURE 3:**
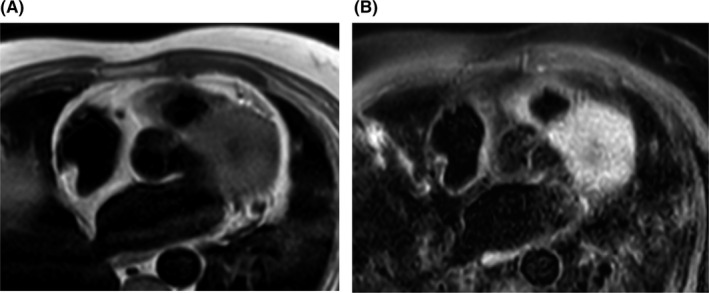
A, T1‐weighted axial stack illustrates uniform signal of the RA structure and the interatrial septum with a signal intensity identical to the subcutaneous fat. B, T1‐weighted fat saturation images illustrate fat suppression on the RA structure and the interatrial septum

**FIGURE 4:**
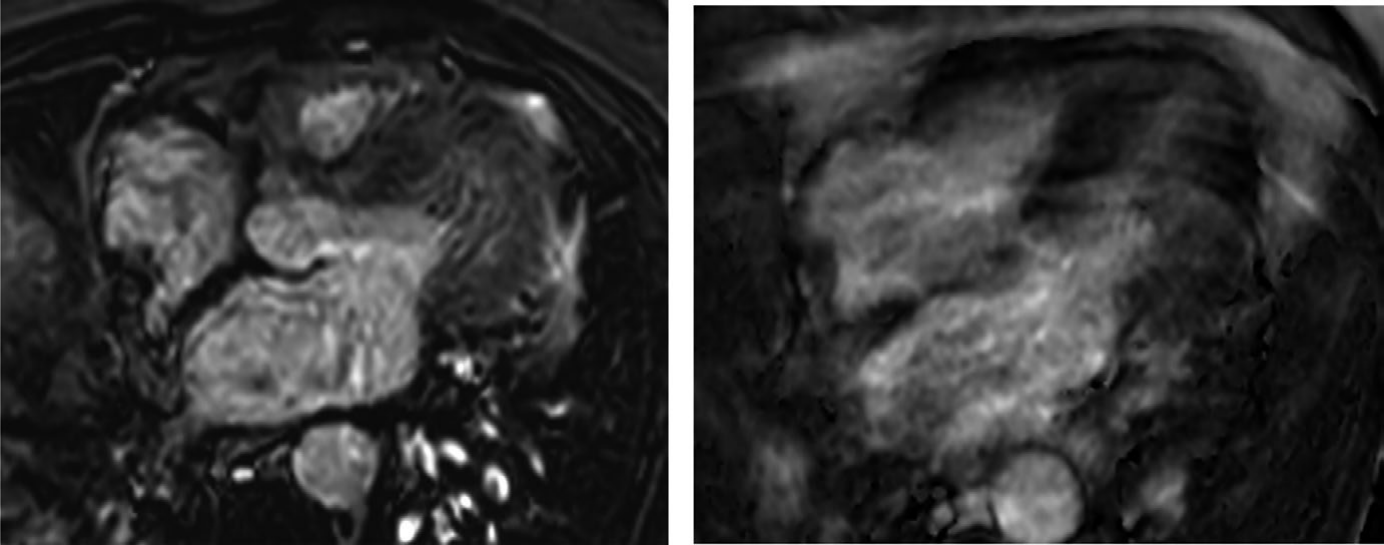
Early (left) and late (right) gadolinium sequences indicated no abnormal enhancement of the mass

The images were obtained on a 1.5 Tesla scanner (Philips Ingenia), and findings from the multiple stacks of cine four‐chamber views confirmed the presence of a structure on the posterior wall of the RA that extends onto the thickened interatrial septum (Figure 2). This smooth structure moved during systole and diastole and was identified as the crista terminalis, which was rather prominent. T1‐ and T2‐weighted images on the axial plane of this structure had a similar signal intensity to the subcutaneous fat revealing it as a predominant fat deposition. T1‐weighted images with fat saturation sequences (Figure 3) demonstrated the suppression of fatty signals consistent with LHIAS of the thickened interatrial septum associated with fatty deposition in the crista terminalis. On performing postgadolinium CMR, early and late acquisition, there were no uptakes or features to suggest the presence of thrombus or any other cardiac tumors (Figure 4).

Cardiac magnetic resonance imaging concluded that the prominent RA mass was lipomatosis hypertrophy of the interatrial septum, which extends to the crista terminalis with no evidence of any thrombus. Hence, the anticoagulation was discontinued, and the patient was discharged home in very good condition.

## 3 DISCUSSION

Lipomatous hypertrophy of the interatrial septum (LHIAS) is usually incidental and typically diagnosed by a transthoracic echocardiography (TTE) by appreciating the classic dumbbell‐shaped morphology within the interatrial septum.^5^ However, an atypical appearance on TTE might happen; therefore, multimodality imaging is required to confirm the diagnosis of LHIAS.

Lipomatous hypertrophy of the interatrial septum typically spares the fossa ovalis while respecting the atrial septum boundaries.^6^ TTE has low sensitivity in diagnosing LHIAS due to limited resolution, especially when the image quality is suboptimal. A transesophageal echocardiogram can be used to easily diagnose LHIAS, which best appears “in the bicaval view” as “globular thickening of the interatrial septum.”

What made matters worse was that this mass was incidentally discovered in this patient with COVID‐19 pneumonia. As has been published in several studies, accelerated thrombosis was frequently reported in patients admitted with COVID‐19. Therefore, in our case, due to severe COVID‐19 symptoms with elevated D‐dimer, the mass was treated as an intracardiac thrombus; initially, heparin and warfarin, then subsequently with warfarin only using a target INR between 2 and 3.

Due to the patient's acute respiratory distress, he was not suitable for any intervention. Therefore, the decision was made not to proceed with TEE to prevent further respiratory deterioration.

Typically, the borders of cardiac tumors are determined by CMR. It also offers a superior tissue characterization. LHIAS follows the same signal intensity of subcutaneous fat.^7^ Sparing the fossa ovalis and extension of greater than 2cm in transverse diameter are unique to LHIAS that differentiates it from lipomas. Typical findings include uniform high signal intensity on T1‐ and T2‐weighted images with signal suppression on fat‐saturated images.^8^ They appear hyperintense also on SSFP cine sequences, with no gadolinium contrast enhancement neither on the first‐pass perfusion nor in the delayed phase.^9^ Other differential here with fatty content would be malignant masses, which demonstrate infiltration of anatomic structures, poor definition of borders, inhomogeneous tissue appearance postgadolinium, and associated pericardial or pleural effusion.^10^ Thus, characterization of localization, morphology, and signal intensity tissue on the CMR helps differentiate between etiologies of tumors in the heart.

## 4 CONCLUSION

This case highlights the importance of understanding RA anatomy, as well as the limitations of TTE, especially in patients with suboptimal image quality. It has also confirmed the importance of the multimodality imaging approach to reach the appropriate diagnosis in such cases.

## CONFLICT OF INTEREST

The authors declare that they have no competing interests.

## AUTHOR CONTRIBUTIONS

FO, ARA, SA, MYK, AJN, MA, AAM, AAQ: Data Collection, Literature Search, Manuscript Preparation. All authors read and approved the final manuscript.

## ETHICS APPROVAL AND CONSENT TO PARTICIPATE

The article describes a case report. Therefore, no additional permission from our Ethics Committee was required.

## CONSENT FOR PUBLICATION

The consent for publication was obtained.

### DATA AVAILABILITY STATEMENT

All data generated or analyzed during this study are included in this published article.
